# Numerical analysis of hafnium oxide and phase change material-based multi-layered infrared and visible frequency sensor for biomolecules sensing application

**DOI:** 10.1038/s41598-023-34817-1

**Published:** 2023-05-11

**Authors:** Khaled Aliqab, Vishal Sorathiya, Meshari Alsharari, Kavan Dave, Ammar Armghan

**Affiliations:** 1 Department of Electrical Engineering, College of Engineering, Jouf University, 72388 Sakaka, Saudi Arabia; 2 Department of Information and Communication Technology, Marwadi University, Rajkot, India; 3 Faculty of Engineering and Technology, Parul Institute of Engineering and Technology, Parul University, Waghodia Road, Vadodara, Gujarat 391 760 India

**Keywords:** Biomedical engineering, Engineering, Materials science, Optics and photonics, Physics

## Abstract

We report on the results of a numerical investigation into a phase transition material and hafnium (IV) oxide-based refractive index sensor with a wide spectral range, including both the visible and infrared regions of the electromagnetic spectrum. The sensor relies on hafnium (IV) oxide and a phase transition material (HfO_2_). Three layered versions of the proposed structure are studied; each configuration is built from alternating layers of HfO_2_, silica, Ge_2_Sb_2_Te_5_(GST), and silver. The three different arrangements have all been studied. The reflectance response of such multilayer structures is discussed in this manuscript for refractive indices ranging from 1 to 2.4. In addition, we have investigated how the varying heights of the materials affect the overall performance of the structure. Finally, we have supplied several formulae for resonating traces that may be used to calculate the sensing behaviour across a specific wavelength range and refractive index values. The corresponding equations are shown below. We have computed numerous equation traces throughout this inquiry to calculate the wavelength and refractive index values. Computational methods may be used to analyze the proposed structure, which might aid in creating biosensors for detecting a wide variety of biomolecules and biomarkers, such as saliva-cortisol, urine, glucose, cancerous and cancerous, and hemoglobin.

## Introduction

Food safety, disease diagnosis, medicine selection, and enzyme detection are areas where biosensors have made great strides in recent years^1,2^. These sensors use all sorts of sensing techniques and equipment. One such method is measuring the index of refraction, which may be used to identify various chemical and biological characteristics. Dissipating charge density oscillations at the dielectric-metal interface are surface plasmons (SPs). A metal's electric field deteriorates at an accelerating rate when exposed to air and water. Stimulating SPs is a potential first step in creating TM-polarized waves from naturally existing materials. Plasmonic devices may use surface plasmon resonance (SPR) as a mechanism^3–5^ to accomplish various chemical and biosensing tasks. The SPR technique allows for the successful execution of such programs. This technology has found use in many areas, including food analysis, drug testing, and medical diagnostics. Because of its numerous advantages, SPR sensors and other contemporary sensing methods are now at the cutting edge of technology for use in sensing applications. The ideal sensor system we could create would be sensitive, quick to respond, and label-free, allowing it to do real-time sensing on any platform. In the paper, the authors use a modified Kretschmann apparatus and attenuated total reflection spectroscopy to excite SPs. In typical Kretschmann's invention, a high-index prism is coated with a thin metal layer^6^. The impingement phenomenon occurs when a TM wave of a certain wavelength comes into contact with a prism at an incidence angle greater than the critical angle between metal and prism at the interface. The metal layer must remain in touch with the dielectric medium being measured. As the energy of an input wave passes through a thin metal layer, it is transformed into a surface plasmon wave in the metal. This results in the creation of what is known as surface plasmon (SP) waves at the interface between a dielectric and metal layer boundaries. This occurs because the wave has to pass through the metal to get to its destination. This takes place because the wave must travel through the metal before reaching its target. The light reflected from the base of a prism is at its weakest when the light enters the prism at a specific angle. The term "resonance angle" is commonly used to refer to this particular angular value. From this, we may deduce that the constants of propagation for evanescent waves and surface-penetrating waves are the same. One of the most important factors when determining this angle is the medium refractive index through which the resonance is generated. A metal layer is often used during the manufacturing process of conventional SPR sensors. Gold (Au)^7^ or silver (Ag)^8^ are typical ingredients in this coating. To construct SPR sensors that are capable of sustaining plasmons, several different metals, such as silver, gold, indium, aluminum, and sodium, are used. Plasmons are even capable of existing in sodium under the appropriate circumstances. A wide range of metals, including copper, silver, indium, gold, aluminum, and sodium, are used to construct SPR sensors capable of sustaining plasmons. Plasmons are theoretically capable of existing in sodium, given the right conditions. Because of its improved stability, biocompatibility, and sensitivity, gold has largely replaced silver as the material of choice for SPR sensors in recent years^9–11^. Historically, silver was often employed in these detectors. One of the many ways gold outperforms silver is through its increased sensitivity. On the other hand, silver can be used to cover an advanced layer, slowing the pace of oxidation in that layer^9–11^. The analyte's relative intensity (RI) before and after contact is compared by researchers as part of their investigation into the impact of biomolecule interactions on sensor sensitivity. For surface plasmon resonance to take place, it is necessary for the evanescent wave generated by the TM light to be in phase with the surface plasmon (SP) wave (SPR). The reflectance profile may go lower if all of these criteria come true. The exact angle at which reflectance starts to decrease is contingent upon several different factors^12–15^. These factors include the kind of prism used, the wavelength of the incident light, the materials, the metal, and the way biomolecules were bound. When evaluating the performance of an SPR sensor in terms of its sensing capabilities, the reflectance curve is the main instrument used for the evaluation. A sensor based on surface plasmon resonance has the potential to identify biomolecules in a liquid sample. Once biomolecules attach to a metal surface, they produce a layer with a higher RI than water. If we analyze a sample, we can see that the resonance angle changes. The degree of adsorption impacts the sensor's ability to identify biomolecules in the presence of background noise. Therefore, while constructing SPR-based sensors, it is essential to consider the type of surface on which biomolecules are adsorbed. The creation of biosensors is highly dependent on fine-tuning, which may be accomplished in part by applying phase transition materials such as GST^16^. Because GST is now a part of the biosensor, it is possible to make more nuanced modifications to the absorber and the sensor. It has been demonstrated^17^ that polarization-insensitive absorbers may be produced by employing GST metasurfaces as the active component. On the other hand, research has shown that GST may boost the performance of plasmonic devices^18^. The most common kind of phase transition material, GST, can flip between an amorphous form (aGST) and a crystalline state (cGST) depending on the circumstances. These states have unique optical and electrical characteristics, making them an appealing material for use in a wide variety of applications, including data storage, sensors, and logical devices^19^. Creating biosensors that may be used in sensing and switching applications can benefit from utilizing modifiable phase change materials. Due to the intense nature of its interaction with light, GST has emerged as a critical component in developing nanophotonic and nanoplasmonic technologies^20^. In contrast to its amorphous condition, the crystalline form of GST can absorb light^17^. When making biosensors using GST, adding gold in the gap between the metal layer and the metal grating results will increased sensitivity for a longer lifespan^21^. Thermally produced silicon nitride (Si_3_N_4_) and silicon dioxide (SiO_2_) have dominated the market for usage as transistor gates in field effect transistors during the past few decades^22,23^. However, conventional biosensors are constructed using semiconducting silicon. When the complementary metal oxide semiconductor (CMOS) thickness with SiO2 material-based devices decreases, high gate oxide leakage becomes more noticeable because the layer's reliability is diminished.

The capacitance may be increased without reducing the dielectric thickness to leaky dimensions by increasing the dielectric constant (K). To find alternatives to SiO_2_ for usage as high-K gate materials, several additional materials have been investigated. The most popular material is silicon dioxide. This category includes tantalum pentoxide (Ta_2_O_5_), titanium dioxide (TiO_2_), zirconium dioxide (ZrO_2_), and hafnium oxide (HfO_2_)^24–26^. HfO2 is one such compound that has been the subject of much research. HfO_2_ is more stable thermally on silicon after atomic layer deposition (ALD) than SiO2. Similarly, Al_2_O_3_ is more stable thermally with Si after ALD^27^. It is a significant difference compared to the other high- K dielectrics. Therefore, HfO_2_ might emerge as a promising high-K gate material. These characteristics might be considered to be the charge impact of the material when used in a biosensor. Because the gate oxide layer produced on the semiconducting channel connects to the channel's consistent capacitance switching behaviour creased thermal stability produces a favorable interface for electrical performance. Applying a high-K material to generate a highly polar surface can help reduce the relative activation energy necessary for surface functionalization. A high-K material can create a highly polar surface to achieve this result.

Hafnium dioxide (HfO_2_) is a metal oxide commonly used in the fabrication of sensors due to its high dielectric constant and excellent electrical properties^28^. In surface plasmon resonance (SPR) sensors, HfO_2_ is often used as a thin film coating to enhance the sensitivity and stability of the sensor. Several polymorphs or crystal structures of HfO_2_ have been identified, including monoclinic, tetragonal, and cubic^29^. However, the most commonly used polymorph for SPR sensor applications is the monoclinic phase, which has a higher dielectric constant and is more stable than the other phases. The choice of HfO_2_ polymorph can significantly impact the performance of an SPR sensor. For example, the monoclinic phase is preferred due to its higher sensitivity and stability, while the tetragonal phase may exhibit lower sensitivity and be more prone to phase transitions in thin films^30^. In the infrared region from 1.2 to 2.5 µm, the Si layer is commonly used as a substrate for various optical and electronic devices. Therefore, hafnium dioxide (HfO_2_) is often used as a thin film coating on Si substrates to fabricate optical and electronic devices for the infrared region. The monoclinic phase of HfO_2_ is generally preferred for use with the Si layer in the infrared region due to its high refractive index and low absorption in this wavelength range. The monoclinic phase of HfO_2_ also exhibits good thermal and mechanical stability, making it suitable for use in high-temperature applications.

### Multi-layered refractive index structure

**Figure 1:**
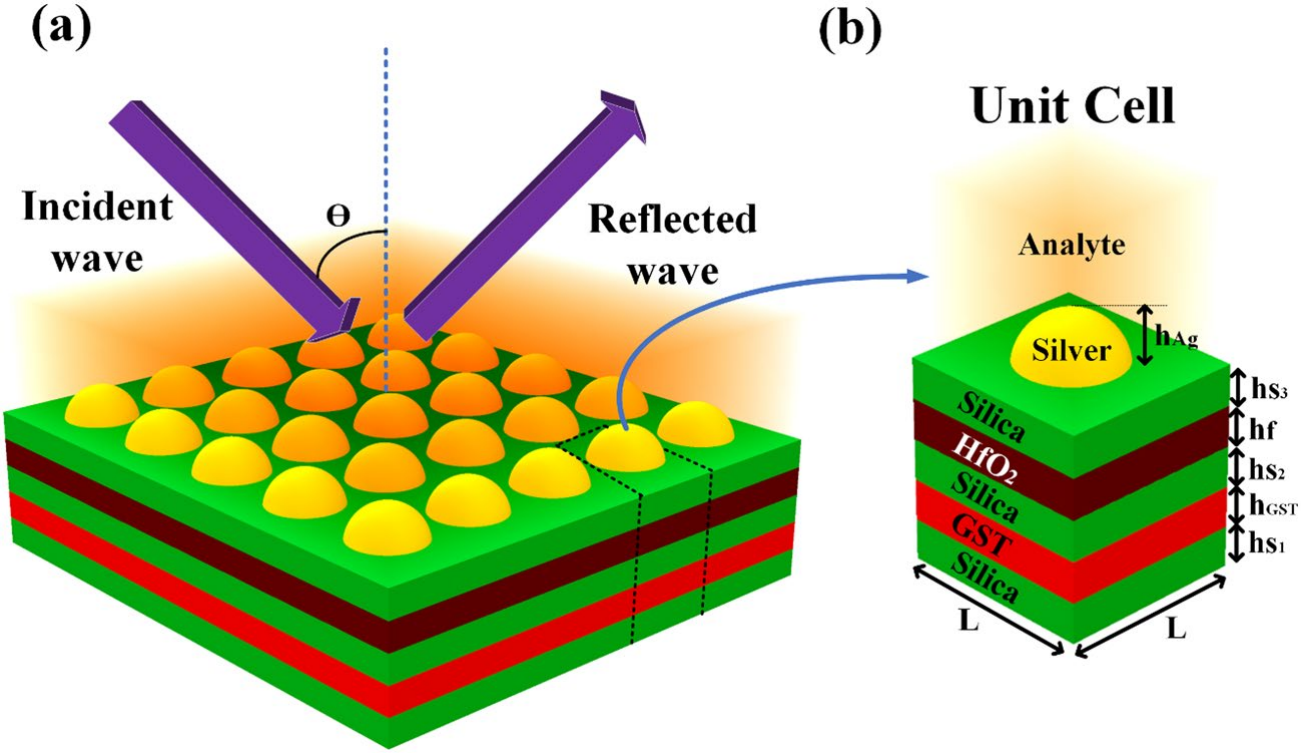
Silver nanoparticles array-based multi-layered tunable refractive index sensor for the infrared frequency spectrum. (**a**)Three-dimensional view of the proposed silver nanoparticle array-based multi-layered material refractive index sensor. (**b**) Unit cell structure with a multi-layered structure formed with Si–GST–Si–HfO_2_–Si–Ag–analyte.

Schematics of the multi-layered Si–GST–Si–HfO_2_–Si–Ag–Analyte-based refractive index sensor are shown in Fig. 1. In this research, a simulation using the finite element technique (FEM) is used to build and evaluate the suggested model. The sensors depicted in Fig. 1 were simulated using COMSOL Multiphysics software. The suggested sensor has been modeled as a 2D model by incident light on top of the multilayer structure (Si–GST–Si–HfO_2_–Si–Ag–Analyte) using periodic boundary conditions and ports in the x and y directions in the two port models^31^. For this FEM-based model, we have used fine physics-controlled scaled mapped mesh with the components ranging from small to large. The infrared wave is excited from the top of the structure, as shown in Fig. 1a. The reflected wave is observed from the same port in reflectance parameters. Port 2 (bottom side of the overall structure in the Z direction) is used to identify the transmittance through the overall unit cell structure.

A unit cell is typically associated with crystalline structures, as it refers to the repeating unit of a crystal lattice^32,33^. However, this does not mean the concept cannot be applied to other structures or systems. In the context of waveguide sensors, defining a unit cell as the basic repeating structure that forms the sensor is possible. Many researchers are using this technique for better understanding. It could include the geometry and dimensions of the waveguide and any materials or coatings used to enhance its sensing capabilities. By understanding the properties of the unit cell, it is possible to predict the behavior of the sensor as a whole. The properties of the metal layer, including its thickness and optical properties, as well as the dimensions and geometry of the waveguide itself, can all be considered part of the unit cell. By understanding how changes in these properties affect the SPR response of the sensor, it is possible to design and optimize the sensor for specific sensing applications. For example, by varying the thickness of the metal layer within the unit cell, it is possible to tune the SPR response of the sensor to different wavelengths of light. It can detect different analytes or molecules in a sample selectively. Similarly, optimizing the geometry of the waveguide within the unit cell can enhance the sensitivity and signal-to-noise ratio of the sensor, leading to improved detection limits and accuracy. Figure 1a shows the proposed three-dimensional view of the proposed structure. Figure 1b shows the unit cell structure with the notation of the dimension. This multi-layered structure is numerically investigated in two-dimensional geometry where the boundary conditions are set periodic boundary conditions. An infrared optical wave is imparted from the top of the proposed structure. The height values are set as h_S1_ = h_S2_ = h_S3_ = 40 nm, h_GST_ = 60 nm, h_f_ = 60 nm and h_Ag_ = 40 nm. The unit cell dimension L is set as 200 nm. We have considered the sandwiched layer of the silica for all the materials because it is easy to grow most of the novel material on a standard silica substrate.

### Resonance condition

The electric field displays a strong discontinuity along the surface normal when free electrons caused by an incident light couple with a metallic surface in contact with a dielectric. It is because the free electrons' motion contradicts the surface normal. Since the E component of s-polarized waves in TE mode is perpendicular to the surface normal, these waves cannot sustain surface plasmons, which are p-polarized (TM mode). Therefore, the electromagnetic components of p-polarized incoming light can be represented as follows (using Eqs. 1 and 2):

1 $${\overrightarrow{E}}_{i}=\left({E}_{{i}_{x}},0,{E}_{{i}_{z}}\right){e}^{i\left({k}_{{i}_{x}}x+{k}_{{i}_{z}}z-\omega t\right)}\,\, \left[\frac{\mathrm{V}}{\mathrm{m}}\right]$$

2 $${\overrightarrow{H}}_{i}=\left(0,{H}_{{i}_{y}},0\right){e}^{i\left({k}_{{i}_{x}}x+{k}_{{i}_{z}}z-\omega t\right)}\,\,[\mathrm{A}/\mathrm{m}]$$

By integrating the preceding equations into Maxwell's equations under suitable boundary conditions^34^, we obtain the equation for establishing resonance, denoted by Eq. (3) below.

3 $$\frac{2\pi }{\lambda }\sqrt{{\varepsilon }_{p}}\mathrm{sin}{\theta }_{RES}=\frac{\omega }{c}\sqrt{\frac{{\varepsilon }_{m}{\varepsilon }_{a}}{{\varepsilon }_{m}+{\varepsilon }_{a}}}$$

In this expression, c represents the velocity of light, λ is the wavelength of the incoming light, the angular frequency is denoted as $\omega$, the incidence angle is denoted as θ_RES_, ${\varepsilon }_{p}$ the permittivity of the prism, ${\varepsilon }_{m}$ that of the metal, and, ${\varepsilon }_{a}$ that of the surrounding medium. The equation above can be reduced to ${k}_{x}=2\pi /{\lambda }_{0}{n}_{p}\mathrm{sin}\theta =\mathrm{Re}\left\{{k}_{SP}\right\}$^35^. In this equation, k_x_ represents the wave vector in the x direction, n_p_ represents the refractive index of the prism, θ represents the incidence angle, λ_0_ represents the wavelength in a vacuum and $\mathrm{Re}\left\{{k}_{SP}\right\}$ specifies the real component of the SP wave vector in the x direction at the metal–dielectric interface. To get the best possible performance, a layer of silicon with a thickness of h_S1=_h _S2=_ h_S3_ = 40 µm. The refractive index of the silicon is determined using the Sellmeir equation, which is written as follows: Eq. (4)

4 $$\begin{array}{l}\\ {n}^{2}\left(Silicon\right)=1+\frac{10.6684293{\lambda }^{2}}{{\lambda }^{2}-(0.301516485{)}^{2}}+\frac{0.003043475{\lambda }^{2}}{{\lambda }^{2}-(1.13475115{)}^{2}} +\frac{1.54133408{\lambda }^{2}}{{\lambda }^{2}-(1104.0{)}^{2}}\end{array}$$

where λ is the wavelength of the incident light and is measured in the µm range. The spectral properties of any specific piece of bulk metal may be characterized by using two different parameters: the plasma wavelength (${\lambda }_{\rm{p}}$) and the bulk collision wavelength (${\lambda }_{\rm{cb}}$). To be more specific, the plasma wavelength is the wavelength that corresponds to the frequency of the electron density oscillations in the metal. Since electron density oscillations are dampened by collisions between electrons in the bulk metal, the wavelength corresponding to these is known as the bulk collision wavelength. The formula for determining the wavelength of plasma is provided in Eq. (5), and it is as follows:

5 $${\lambda }_{\rm{p}}=\frac{1}{\sqrt{\frac{N{e}^{2}}{4{\pi }^{2}{c}^{2}m{\varepsilon }_{0}}}}$$

The formula for calculating the collision wavelength, which is denoted above as Eq. (6), is as follows:

6 $${\lambda }_{\rm{cb}}=\frac{2\pi c{R}_{\text{bulk }}}{{v}_{\rm{f}}}$$

In this equation, $N$ represents the concentration of electrons, $e$ represents the charge of an electron, $c$ represents the speed of light, ${\varepsilon }_{0}$ represents the permittivity of vacuum, ${v}_{\rm{f}}$ represents the velocity of electrons at the Fermi energy, $m$ represents the mass of an electron, and R_bulk_ represents the mean free path of conduction electrons at the Fermi energy. It is necessary to examine a straightforward and accurate model to get the best possible outcome when dealing with the conductivity of the metal. The Lorentz–Drude model is a method to understand the electromagnetic characteristics of metals grounded in classical mechanics. This model is built on three essential assumptions to identify metals' conductivity. This method results in an accurate representation of metals such as gold, silver, and aluminum. The Lorentz–Drude model is the most practical choice when parameterizing the metal's optical constants^36^. Both bound and free electrons affect the optical properties typical of metallic media. As a result, the Lorentz term for the interband ransition and the Drude component for the intraband effect are both accounted for in the form of the Drude–Lorentz model inside the complex dielectric permittivity that corresponds to them ^37^. According to the free electron Drude model, the complex dielectric constant of the metal may be represented in terms of the plasma and collision wavelength using the formula provided in Eq. (7)^38^. It was found out by expressing it in terms of the plasma wavelength.

7 $${\varepsilon }_{\rm{m}}(\lambda )={\varepsilon }_{\rm{mr}}+\mathrm{i}{\varepsilon }_{\rm{mi}}=1-\frac{{\lambda }^{2}}{{\lambda }_{\rm{p}}^{2}\left(1+\mathrm{i}\frac{\lambda }{{\lambda }_{\rm{cb}}}\right)}$$

where λ is a specific wavelength among the range of wavelengths that have been targeted, λ_p_ is the wavelength of the plasmonic resonance, and ${\lambda }_{\rm{cb}}$ is the wavelength at which the two waves collide. The values 1.4541 × 10^–7^ m and 1.7614 × 10^−5^ m have been extracted from data available in ^39,40^. For the plasmonic and collision wavelengths, respectively, for the proper wavelength range and specific silver metal. These values were derived. The equation used to get the index of refraction when starting with this information is given in Eq. (8).

8 $$\mathrm{n_{Ag}}(\lambda )=\sqrt{1-\frac{{\lambda }^{2}{\lambda }_{c}}{{\lambda }_{p}^{2}\left({\lambda }_{cb}-i\lambda \right)}}$$

The refractive indices of PCM, such as aGST and cGST, were calculated as the function of frequency. The real part of aGST is in the range of 2.6 to 4.6 and the imaginary part is in the range of 0 to 2.4 for the range of 100 to 800 THz. Similarly, the real part of cGST is in the range of 2.25 to 7.16, and the imaginary part is from 0 to 4.1 in the range of 100 to 800 THz. The relative data is considered from the data available in^41^.

### Numerical analysis of the proposed biosensor

After observing the electrical distribution from the FEM method in COMSOL physics software, radiative properties of the biosensor's multilayers, such as reflectance and transmittance, may be calculated using one of three methods: the transfer matrix method, the field tracing methodology, or the resulting wave method. The transfer matrix approach is the most precise of these methods since it does not rely on approximations. Thus, we will apply the transfer matrix method (TMM) on the biosensor to study the performance characteristics of the suggested multilayer structure for parallel polarisation light arriving from the top of the z-axis in the two-port model, such as reflectance. It was done so that we could learn more about these features. We apply the boundary conditions indicated in Eq. (9)^42^, resulting in the following matrix equation, representing the interrelationship between the tangential electric field and the magnetic field components at the first-layer boundary and the last-layer boundary, respectively.

9 $$\left[\begin{array}{l}{E}_{1}\\ {H}_{1}\end{array}\right]=T\left[\begin{array}{c}{E}_{N-1}\\ {H}_{N-1}\end{array}\right]$$

10 $${T}_{ij}={\left(\prod_{m=2}^{N-1} {T}_{m}\right)}_{ij}=\left[\begin{array}{c}{T}_{11}\hspace{0.25em}\hspace{0.25em}\hspace{0.25em}\hspace{0.25em}{T}_{12}\\ {T}_{21}\hspace{0.25em}\hspace{0.25em}\hspace{0.25em}\hspace{0.25em}{T}_{22}\end{array}\right] \quad for \,\,\, i,j=\mathrm{1,2},\dots$$

E1 and E_N-1_ are electric field components, and H_1_ and H_N-1_ are magnetic field components for layers 1 and N, respectively. Equation (10) represents the further simplified T matrix^43^. To build the transfer matrix, it is necessary to calculate the phase shift and admittance values for each layer using the formula presented in Eq. (11) ^44^.

11 $${\beta }_{m}=\frac{2\pi }{\lambda }{d}_{m}\sqrt{{n}_{m}^{2}-{\left({n}_{p}\mathrm{sin}\left({\theta }_{in}\right)\right)}^{2}},{q}_{m}=\frac{\sqrt{{n}_{m}^{2}-{\left({n}_{p}\mathrm{sin}\left({\theta }_{in}\right)\right)}^{2}}}{{n}_{m}^{2}}$$

where q_m_ and β_m_ are the admittance and phase shift of the mth layer, respectively, to determine these, it is necessary to know specific parameters, such as n_m_, which stands for the refractive index of the mth layer, d_m_, which refers to the thickness of the mth layer, n_p_, which stands for the refractive index of the prism, and in, which stands for the incident angle of the prism. For the N-layer surface plasmon sensor, it is necessary to consider the aggregate of these reflections when calculating total reflection because different reflections arise at the interface of each layer depending on the incoming light at the prism and the first layer. These reflections are dependent on one another. In the N-layer model, a P-polarized propagating wave through the successive layers can be characterized by the Transfer matrix, as given in Eq. (12). On further mathematical simplifications, the reflection coefficient for the p-polarized incident light in N-layer proposed biosensor is calculated as Eq. (13).

12 $${T}_{m}=\left[\begin{array}{cc}cos\left({\beta }_{j}\right)& -\mathrm{i sin}\left({\beta }_{j}\right)/{q}_{j}\\ -i{q}_{j}\mathrm{sin}\left({\beta }_{j}\right)& \mathrm{cos}\left({\beta }_{j}\right)\end{array}\right]$$

13 $${r}_{p}={\left|\frac{\left.\langle {T}_{11}+{T}_{12}{q}_{N}\right){q}_{1}-\left({T}_{21}+{T}_{22}{q}_{N}\right)}{\left({T}_{11}+{T}_{12}{q}_{N}\right){q}_{1}+\left({T}_{21}+{T}_{22}{q}_{N}\right)}\right|}^{2}$$

**Figure 2:**
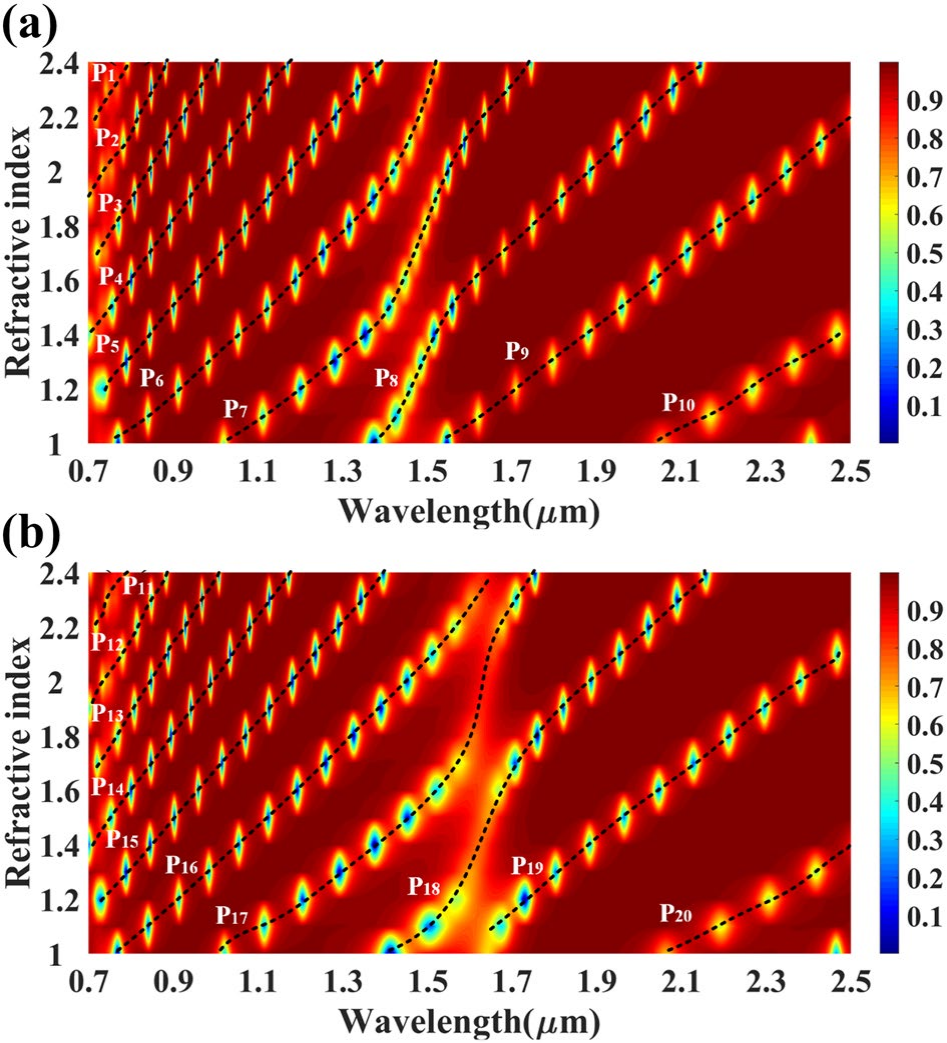
Reflectance response for the different (**a**) aGST phase and (**b**) cGST phase of the phase change material. The traces marked as P_1_ to P_20_ are the possible quadratic equation for identifying the sensitivity for the specific range of the refractive index and wavelength. The layered structure formed in this response is analyte–Ag–Si–HfO_2_–Si–GST–Si.

**Figure 3:**
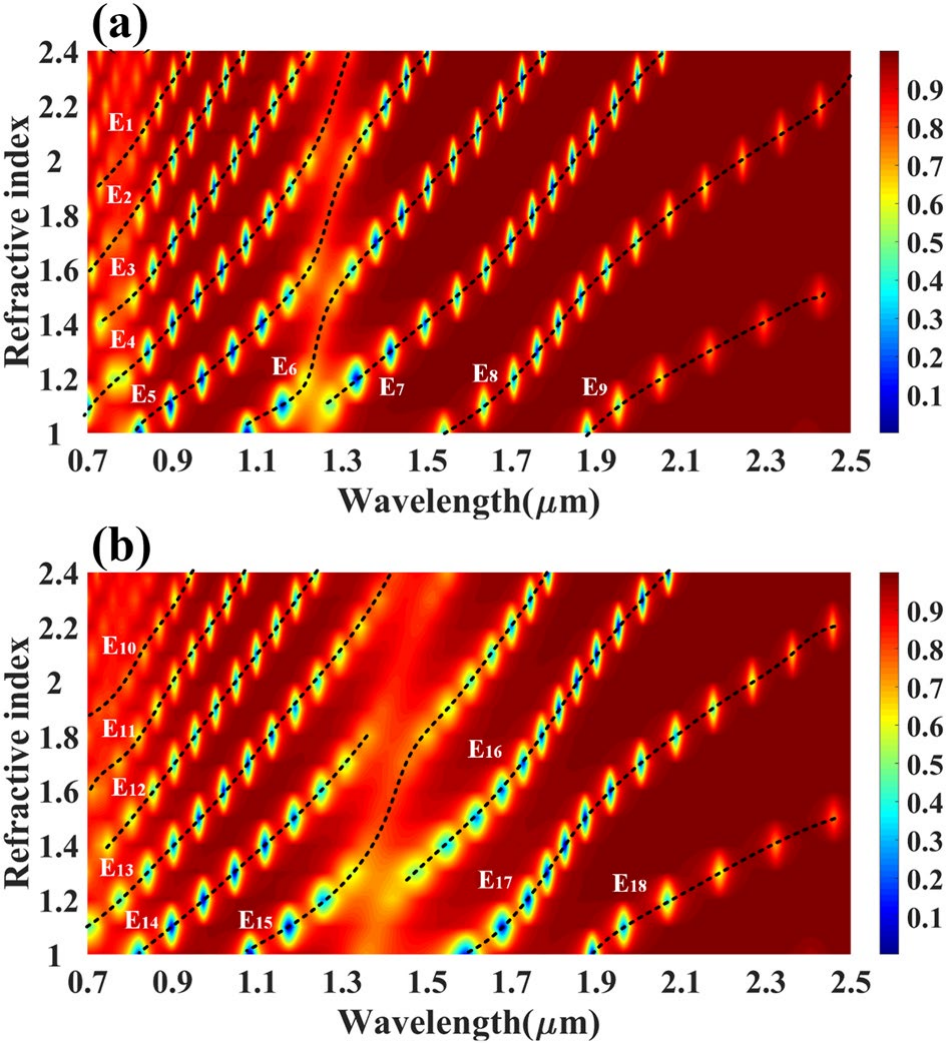
Calculated reflectance response for the (**a**) aGST phase and (**b**) cGST phase of GST in the absence of HfO_2_ material. The layered structure formed in this response is analyte–Ag–Si–GST–Si. In addition, a proposed quadratic equation for determining the sensitivity across a given range of refractive index and wavelength is shown by the E_1_–E_18_ traces.

At last, the reflectance of the multilayer structure is expressed as ${R}_{p}={\left|{r}_{p}\right|}^{2}.$ We have numerically investigated the proposed multi-layered structure using COMSOL multiphysics software. Initially, we set the height values of all the structures as 40 nm. The calculated reflectance values of the reflectance port for the different refractive index values are shown in Fig. 2. Figure 2a shows the variation in the reflectance for the analyte–Ag–Si–HfO_2_–Si–GST–Si layered structure by considering the aGST as a phase of the GST for RI values of 1 to 2.4. Similarly, Fig. 2b shows the calculated variation in the proposed structure's reflectance for the cGST phase. In both figures, we have identified a total of 20 traces equation (P_1_–P_20_) of the minimum reflectance values. The notable wavelength shift on both phases of the GST material is also observed in the overall spectral response. The effect of the HfO_2_ layer on the overall reflectance spectrum for the RI values of 1 to 2.4 is also investigated and presented in Fig. 3a, b. Figure 3a shows the reflectance variation for the entire wavelength spectrum and refractive index spectrum for analyte–Ag–Si–GST–Si structure under aGST phased condition of GST material. Similarly, Fig. 3b shows the variation in the reflectance for the cGST phase conditions of GST material for analyte–Ag–Si–GST–Si layered structure. We have identified the total 18 reflectance traces (E_1_ to E_18_) for the specific values of wavelength and refractive index ranges. The derived traces with their wavelength range, refractive index range, and a quadratic equation in Table 1 for the analyte–Ag–Si–HfO_2_–Si–GST–Si layered structure. Similarly, the derived quadratic equation for the analyte–Ag–Si–GST–Si-based structure is shown in Table 2. We can use this equation to design the biosensor where the different refractive index analytes show different resonance peaks at different wavelength values. This sensor can be utilized in the creation of infrared biosensors for detecting ethanol, water, glucose, urine^45^, biotin-streptavidin, fibrinogen ^46,47^, and hemoglobin ^46,47^. The value of the refractive index of this biomolecule is majorly ranging from 1 to 1.7 depending on the concertation of the biomarkers.

**Table 1:** Derived fitting equations for the different traces found in aGST and cGST phase of the proposed analyte–Ag–Si–HfO_2_–Si–GST–Si structure for different wavelength and refractive index ranges.

Design	Trace	Wavelength range ($\mathrm{\lambda in \mu m})$	Refractive index $(\mathrm{n})$	Fitting equation
	E1	(0.72, 0.93)	(1.91,2.38)	$\uplambda =-24.5264{\mathrm{n}}^{3}+63.7145{\mathrm{n}}^{2}-52.5753\mathrm{n}+15.8852$
	E2	(0.78. 1.06)	(1.76,2.40)	$\uplambda =-6.0189{\mathrm{n}}^{3}+15.0025{\mathrm{n}}^{2}-10.1467\mathrm{n}+3.3914$
	E3	(0.73,1.21)	(1.41, 2.38)	$\uplambda =-2.6547{\mathrm{n}}^{3}+8.4055{\mathrm{n}}^{2}-6.6480\mathrm{n}+2.8008$
	E4	(0.69,1.31)	(1.31, 2.38)	$\uplambda =4.9489{\mathrm{n}}^{3}-13.3718{\mathrm{n}}^{2}+13.5039\mathrm{n}-3.5263$
	E5	(0.81,1.49)	(1.01, 2.38)	$\uplambda =-27.9849{\mathrm{n}}^{5}+129.8418{\mathrm{n}}^{4}-225.8289{\mathrm{n}}^{3}+179.7946{\mathrm{n}}^{2}-60.5107\mathrm{n}+5.9265$
	E6	(1.0,1.77)	(1.03, 2.38)	$\uplambda =-117.3662{\uplambda }^{5}+858.2018{\uplambda }^{4}-2492.0735{\mathrm{n}}^{3}+3590.3699{\mathrm{n}}^{2}-2563.1039\mathrm{n}+725.5755$
	E7	(1.26,2.05)	(1.11, 2.38)	$\uplambda =-0.3495{\mathrm{n}}^{3}+2.5379{\mathrm{n}}^{2}-3.8442\mathrm{n}+2.6359$
	E8	(1.54,2.5)	(1, 2.36)	$\uplambda =-0.2061{\mathrm{n}}^{3}+0.7432{\mathrm{n}}^{2}+0.9396\mathrm{n}-1.5120$
	E9	(1.89,2.43)	(1.01, 1.50)	$\uplambda =0.6666{\mathrm{n}}^{3}-4.7387{\mathrm{n}}^{2}+12.0010\mathrm{n}-9.2353$
	E10	(0.70,0.94)	(1.87, 2.40)	$\uplambda =-19.3064{\mathrm{n}}^{3}+50.1595{\mathrm{n}}^{2}-40.9114\mathrm{n}+12.5501$
	E11	(0.71,1.06)	(1.62, 2.40)	$\uplambda =-10.8119{\mathrm{n}}^{3}+30.1545{\mathrm{n}}^{2}-25.5130\mathrm{n}+8.4103$
	E12	(0.86,1.23)	(1.61, 2.40)	$\uplambda =-0.3250{\mathrm{n}}^{3}+1.1931{\mathrm{n}}^{2}+0.6615\mathrm{n}+0.3679$
	E13	(0.69,1.40)	(1.10, 2.38)	$\uplambda =-0.1467{\mathrm{n}}^{3}+1.0458{\mathrm{n}}^{2}+0.0935\mathrm{n}+0.5689$
	E14	(0.82,1.35)	(1, 1.79)	$\uplambda =1.1638{\mathrm{n}}^{3}-3.2300{\mathrm{n}}^{2}+4.2928\mathrm{n}-0.9897$
	E15	(1.07,1.78)	(1.01, 2.40)	$\uplambda =95.3522{\mathrm{n}}^{5}-673.2390{\mathrm{n}}^{4}+1881.7529{\mathrm{n}}^{3}-2600.8871{\mathrm{n}}^{2}+1778.6185\mathrm{n}-480.8560$
	E16	(1.45,2.06)	(1.27, 2.4)	$\uplambda =-3.3578{\mathrm{n}}^{3}+18.3768{\mathrm{n}}^{2}-31.3586\mathrm{n}+18.3599$
	E17	(1.60,2.46)	(1.01,2.20)	$\uplambda =0.2730{\mathrm{n}}^{3}-2.4724{\mathrm{n}}^{2}+8.0646\mathrm{n}-6.7459$
	E18	(1.84,2.45)	(1.01,1.49)	$\uplambda =-0.0147{\mathrm{n}}^{3}-0.2512{\mathrm{n}}^{2}+2.1507\mathrm{n}-2.0526$

**Table 2:** Derived fitting equations for the different traces found in aGST and cGST phase of the proposed analyte – Ag – Si – GST – Si structure for a specific range of wavelength and refractive index range.

	P1	(0.71, 0.79)	(2.19, 2.40)	$\uplambda =196.4835{\mathrm{n}}^{3}-446.6786{\mathrm{n}}^{2}+340.7917\mathrm{n}-84.9474$
	P2	(0.70, 0.88)	(1.91, 2.40)	$\uplambda =12.5837{\mathrm{n}}^{3}-26.1819{\mathrm{n}}^{2}+20.3121\mathrm{n}-3.7931$
	P3	(0.72, 1.00)	(1.70, 2.40)	$\uplambda =0.6853{\mathrm{n}}^{3}-1.6957{\mathrm{n}}^{2}+3.9065\mathrm{n}-0.4966$
	P4	(0.70, 1.17)	(1.41, 2.40)	$\uplambda =-0.6806{\mathrm{n}}^{3}+1.9507{\mathrm{n}}^{2}+0.3151\mathrm{n}+0.4486$
	P5	(0.74, 1.38)	(1.20, 2.40)	$\uplambda =0.3521{\mathrm{n}}^{3}-1.0162{\mathrm{n}}^{2}+2.7789\mathrm{n}-0.4368$
	P6	(1.03, 1.73)	(1.01, 2.40)	$\uplambda =-2.1064{\mathrm{n}}^{5}-14.1197{\mathrm{n}}^{4}+115.1238{\mathrm{n}}^{3}-254.6902{\mathrm{n}}^{2}+234.1985\mathrm{n}-77.5072$
	P7	(0.76, 1.52)	(1.02, 2.38)	$\uplambda =2.9695{\mathrm{n}}^{3}-9.1534{\mathrm{n}}^{2}+10.6643\uplambda -3.1543$
	P8	(1.37, 2.14)	(1, 2.38)	$\uplambda =3.0272{\mathrm{n}}^{3}-16.9972{\mathrm{n}}^{2}+33.1601\mathrm{n}-20.3804$
	P9	(1.55, 2.49)	(1.02, 2.2)	$\uplambda =-0.0377{\mathrm{n}}^{3}+0.3166{\mathrm{n}}^{2}+0.4347\mathrm{n}-0.2800$
	P10	(2.05, 2.47)	(1.02, 1.39)	$\uplambda =-2.7129{\mathrm{n}}^{3}+18.5592{\mathrm{n}}^{2}-41.3140\mathrm{n}+31.0885$
	P11	(0.71, 0.78)	(2.21, 2.39)	$\uplambda =-467.7488{\mathrm{n}}^{3}+1034.3394{\mathrm{n}}^{2}-758.9448\mathrm{n}+187.0490$
	P12	(0.7, 0.78)	(1.92, 2.39)	$\uplambda =-2.6489{\mathrm{n}}^{3}+8.2162{\mathrm{n}}^{2}-5.3892\mathrm{n}+2.5737$
	P13	(0.72, 1)	(1.69, 2.39)	$\uplambda =-2.1243{\mathrm{n}}^{3}+5.0438{\mathrm{n}}^{2}-1.4500\mathrm{n}+0.9103$
	P14	(0.71, 1.17)	(1.40, 2.39)	$\uplambda =0.8281{\mathrm{n}}^{3}-2.1708{\mathrm{n}}^{2}+3.9809\mathrm{n}-0.6176$
	P15	(0.73, 1.4)	(1.19, 2.4)	$\uplambda =-0.0900{\mathrm{n}}^{3}+0.3298{\mathrm{n}}^{2}+1.4211\mathrm{n}+0.0151$
	P16	(0.76, 1.63)	(1, 2.4)	$\uplambda =0.3764{\mathrm{n}}^{3}-1.0674{\mathrm{n}}^{2}+2.4064\mathrm{n}-0.3860$
	P17	(1, 1.74)	(1.1, 2.4)	$\uplambda =-634.9904{\mathrm{n}}^{7}+5641.9155{\mathrm{n}}^{6}-21254.9414{\mathrm{n}}^{5}+43995.1875{\mathrm{n}}^{4}-54010.3568{\mathrm{n}}^{3}+39302.6521{\mathrm{n}}^{2}-15684.9115\mathrm{n}+2646.4393$
	P18	(1.41, 2.1)	(1, 2.38)	$\uplambda =-42.6147{\mathrm{n}}^{5}+404.5183{\mathrm{n}}^{4}-1525.9334{\mathrm{n}}^{3}+2857.4420{\mathrm{n}}^{2}-2652.6913\mathrm{n}+976.6201$
	P19	(1.65, 2.47)	(1.09, 2.1)	$\uplambda =0.1174{\mathrm{n}}^{3}-0.8905{\mathrm{n}}^{2}+3.3921\mathrm{n}-2.6149$
	P20	(2.1, 2.5)	(1, 1.4)	$\uplambda =2.5618{\mathrm{n}}^{3}-16.9260{\mathrm{n}}^{2}+38.0244\mathrm{n}-27.9003$

**Figure 4:**
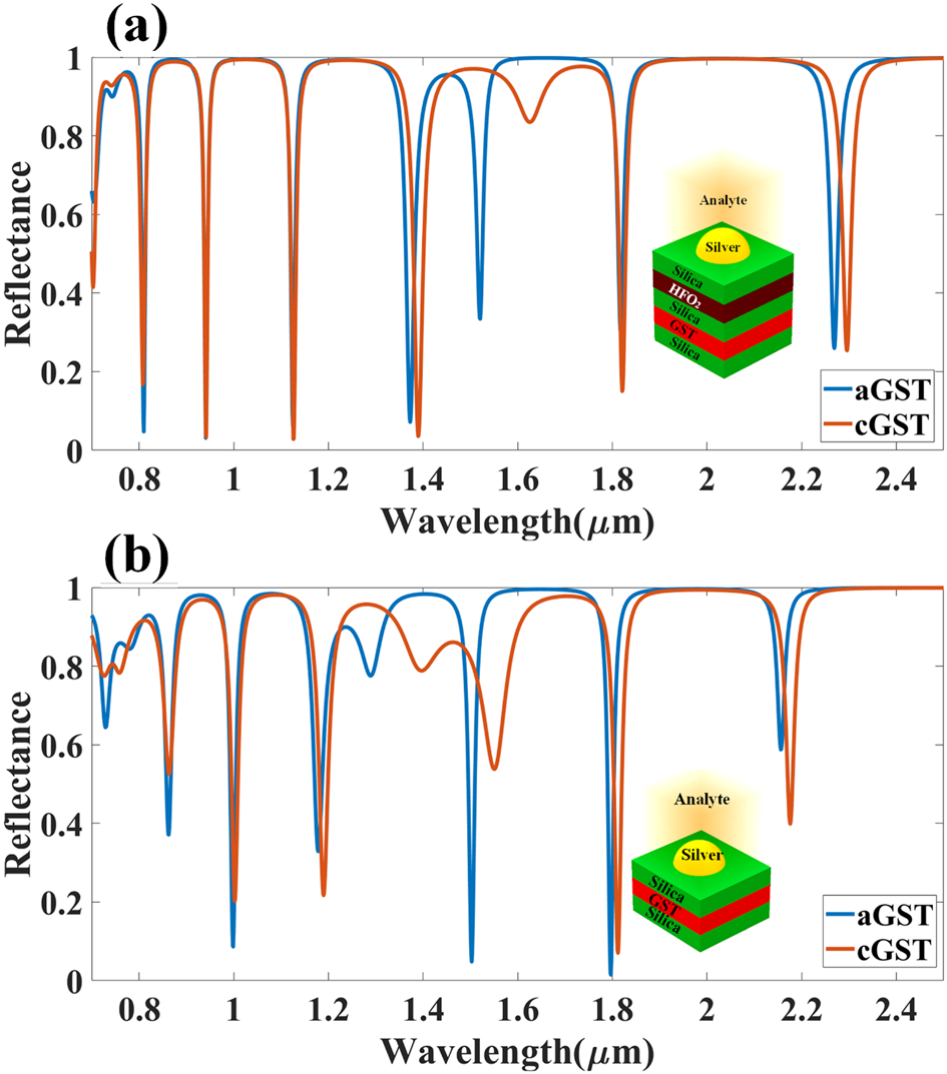
Comparative analysis of the reflectance response generated (**a**) with HfO_2_ and (**b**) without HfO_2_ layer-based SPR refractive index sensor. The variation in both layered structures is shown for the phase change material's two phases (aGST/cGST).

**Figure 5:**
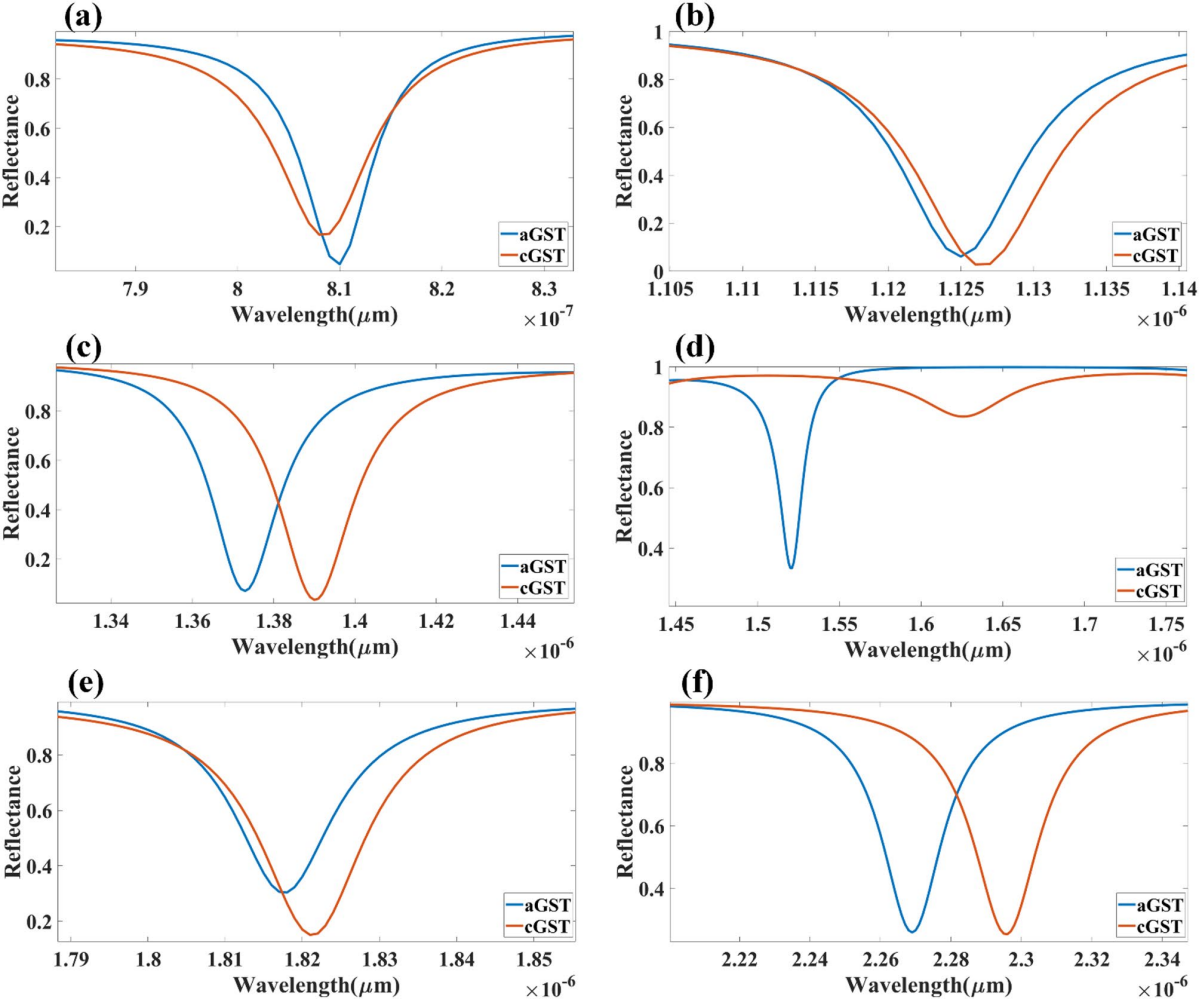
(**a–f**) Comparative plots to identify the wavelength shift while the phase change material will change its state from amorphous to crystalline (aGST to cGST). The response is presented for the specific range of the wavelength where the reflectance values are minimum. The response is generated for the structure with layers of SPR structure as analyte–Ag–Si–HfO_2_–Si–GST–Si.

**Figure 6:**
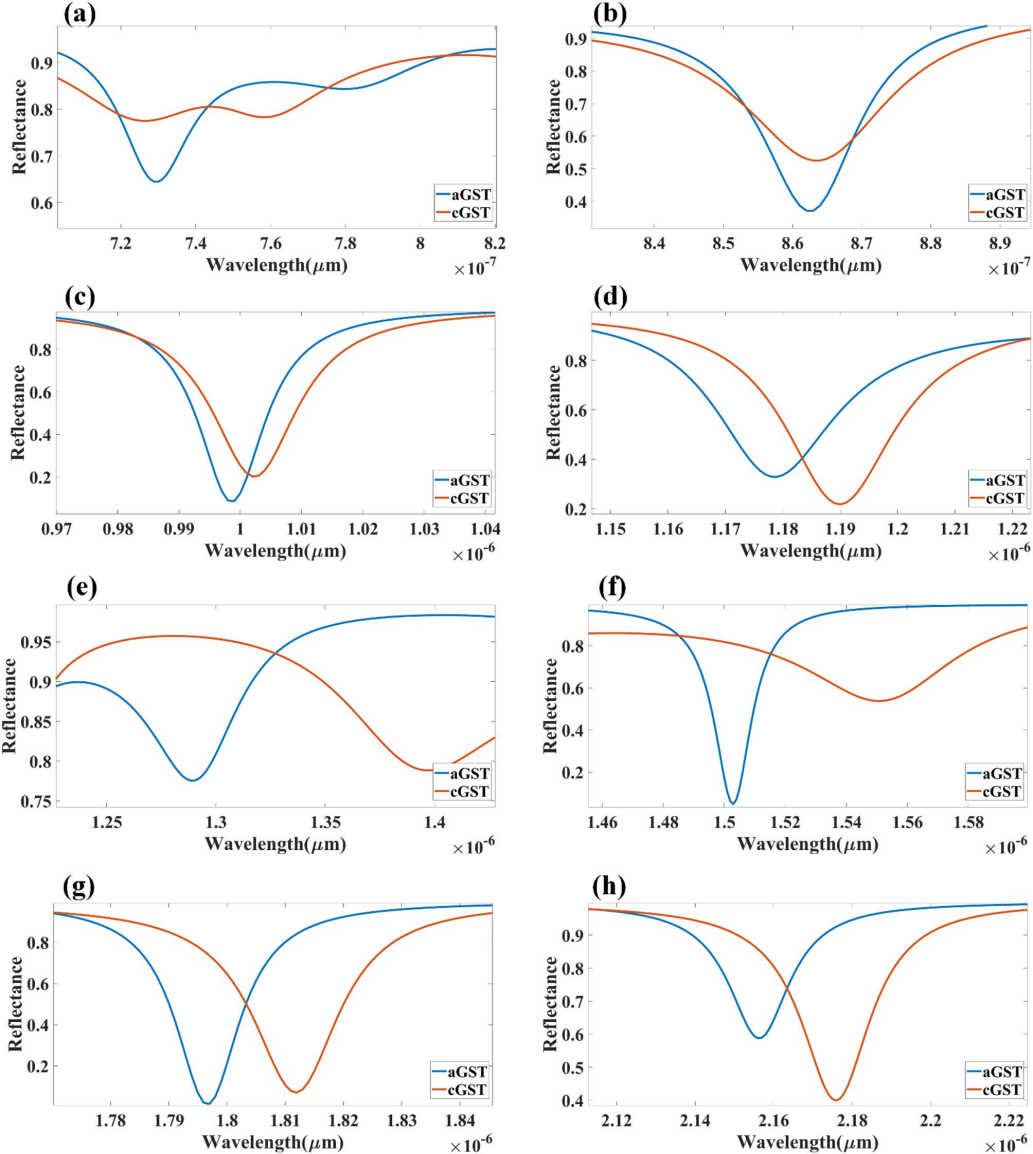
(**a–h**) Comparative plots to identify the wavelength shift while the phase change material will change its state from amorphous to crystalline (aGST to cGST). The response is presented for the specific range of the wavelength where the reflectance values are minimum. The response is generated for the structure with layers of SPR structure as analyte–Ag–Si–GST–Si.

**Figure 7:**
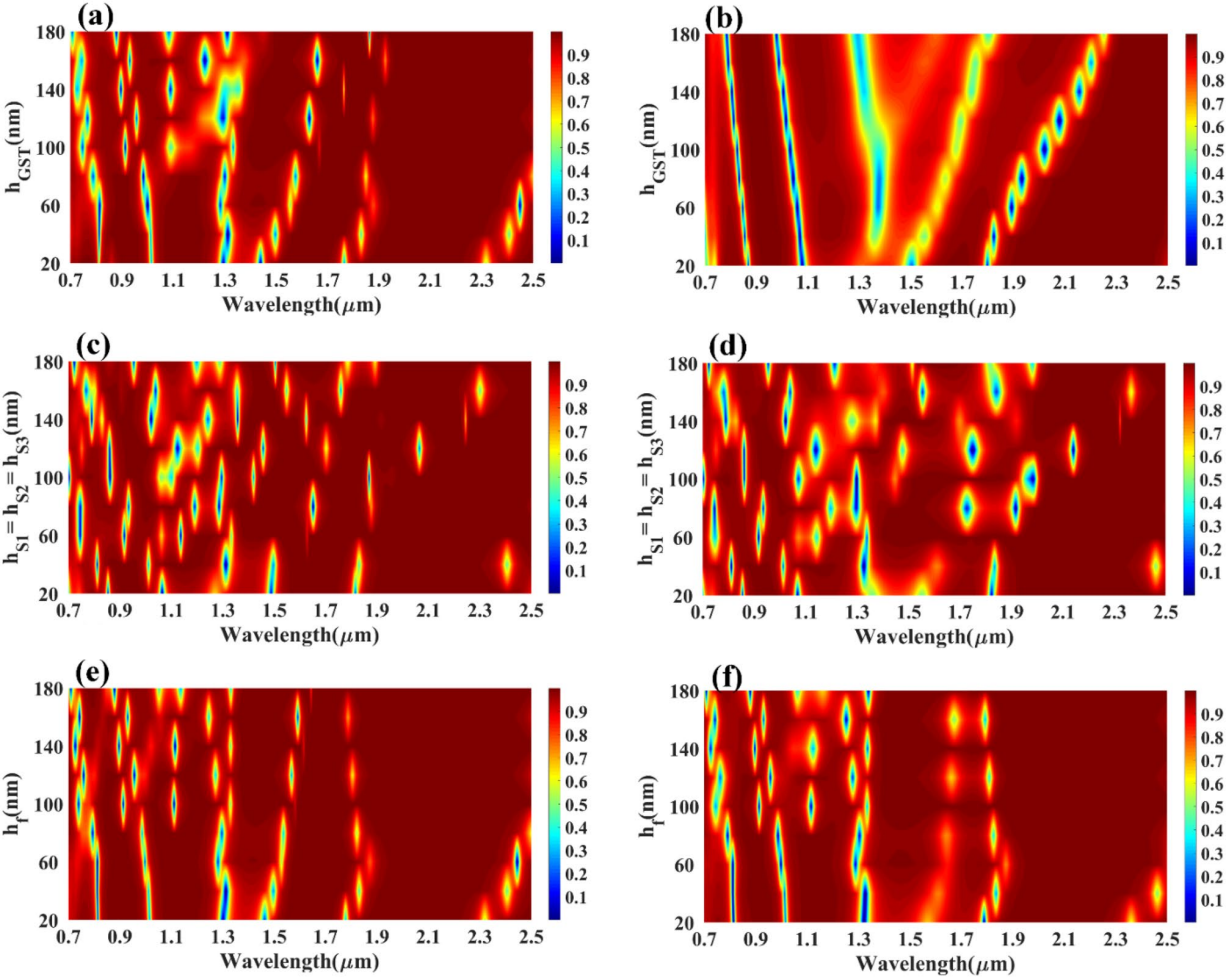
Calculated reflectance response for the different heights of material layers. Reflectance variation for different height values of the (**a**) GST, (**c**) silica, and (**e**) HfO_2_ for the aGST phase of the phase change material. Reflectance variation for different height values of the (**b**) GST, (**d**) silica, and (**f**) HfO_2_ for the cGST phase of the phase change material.

**Figure 8:**
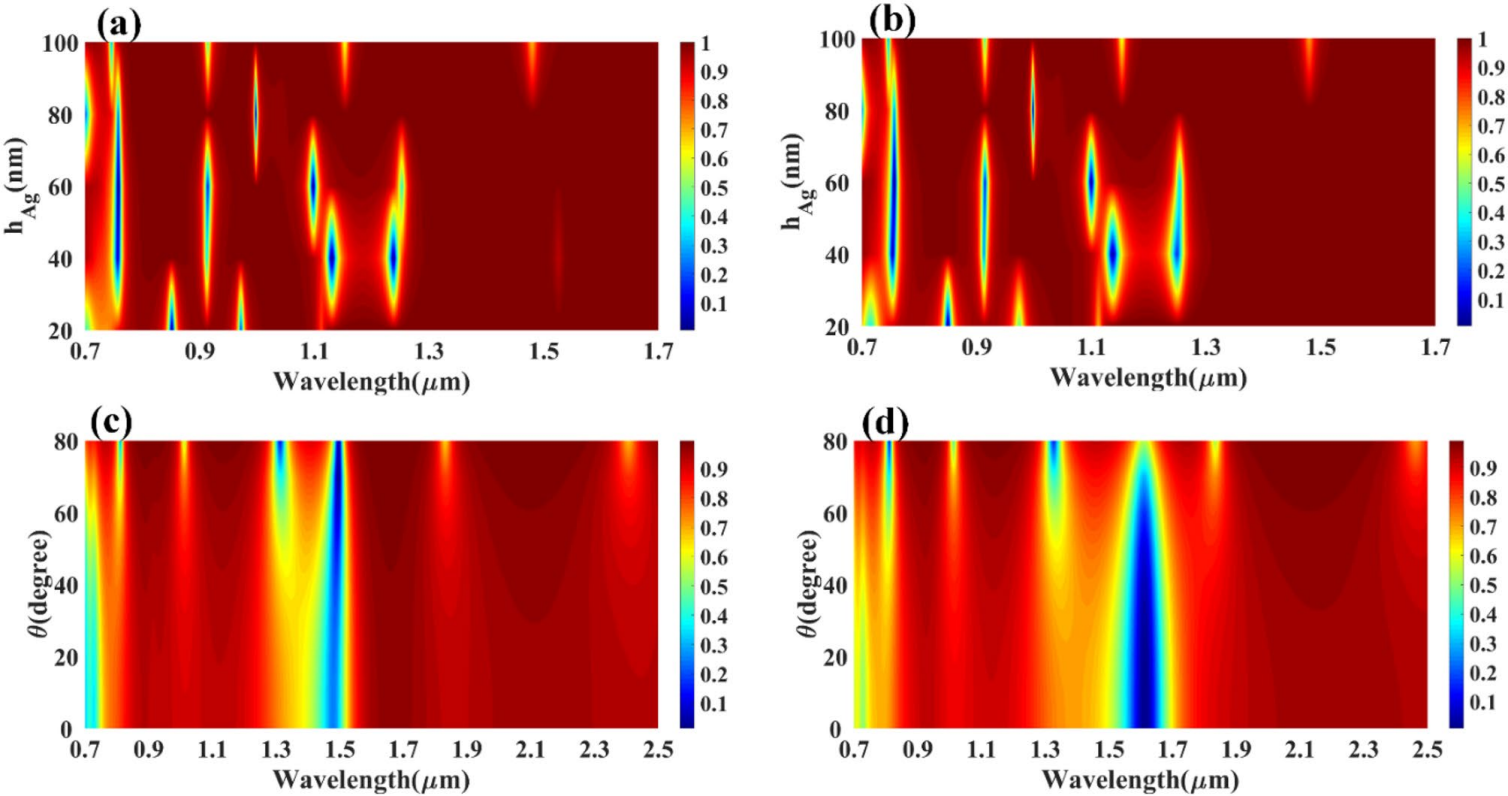
Calculated reflectance response for the different values of the resonator height (h_Ag_) for (**a**) aGST and (**b**) cGST phase of the GST material. The effect in the reflectance while a change in the incident angle of the input waves for (**a**) aGST and (**b**) cGST of the phase change material.

The comparative analysis of different layered structures is shown in Fig. 4 for the HfO_2_–GST and only GST-based structures. The change in reflectance for the calculated infrared spectrum for the HfO_2_–GST material structure is shown in Fig. 4a. Similarly, the reflectance structure for only the GST-based structure may be shown in Fig. 4b. The refractive index for this calculation is set as 2.1 for both plots. The detailed wavelength shift for both structures is shown in Figs. 5 and 6. We have highlighted the six wavelength shift plots for HfO_2_–GST structure and eight only GST-based structure. In HfO_2_–GST-based structure, the maximum wavelength shift of 100 nm is observed between 1.52 and 1.62 µm of the band, as shown in Fig. 5d. The minimum wavelength of 1 nm is observed between 0.79 to 0.83 µm band of operation. Similarly, a maximum wavelength shift of 110 nm is observed between 1.25 to 1.4 µ of the wavelength spectrum for only the GST-based refractive index sensor, as shown in Fig. 6e. The minimum wavelength shift of 20 nm is observed between the 8.4 to 8.9 µm band, as shown in Fig. 6b. Overall it is observed that the change in the phase of the GST material (aGST–cGST) makes the overall wavelength shift over the calculated wavelength spectrum, which ultimately results in the tunability of the overall refractive index sensor. The temperature of the GST material can be controlled using an external thermal source, such as an integrated heater structure, to achieve the tunability of these photonics devices. The effect of various physical parameters on the reflectance behavior of the sensor is calculated and presented in Figs. 7 and 8. Figure 7a, b show the variation in reflectance for the different phases of the GST material and values of the GST height. It is identified that there is a significant dependency on the height of GST material. The varied scattered response in aGST phase is also observed for the different height values. The values of the silica and HfO_2_ are kept at 40 nm for this calculation. We can observe the positive and negative slope of the wavelength and GST height, as shown in Fig. 7b. The effect of GST height is more significant for the > 1.3 µm of the wavelength spectrum. The effect of the silica height on the refraction performance is shown in Fig. 7c, d. Figure 7c, d show the variation in reflection amplitude for aGST/cGST phase, respectively. The variation in Silica height allows us to choose the wafer for the development of the upper layer growth of GST/HfO_2_/Ag. Similarly, the effect of the HfO_2_ layer is shown in Fig. 7e, f for the aGST and cGST structure, respectively. In both silica and HfO_2_, the reflection values are majorly dependent on height due to the light trapping intensity by these layers.

The top Ag resonator will generate the dipole moment in the proposed refractive index sensor to trap the light. Figure 8a, b, respectively, indicate the influence on reflectance for the varied height values of the silver resonators for the aGST phase and the cGST phase of the material. In this response, it has been noticed that there are some subtle differences between the aGST phase and the cGST phase. While changes in height for individual phase also shows minor changes for these parameters. In some of the wavelength points, the resonating wavelength is not shifted much compared to other physical parameter changes. The effect of the oblique angle incident on the overall performance of the reflectance is shown in Fig. 8c, d. It is observed that the angle depended on the response for the specific resonating points. In Fig. 8c, the constant reflectance response is observed at 1.5 µm for the aGST phase of the GST material. Similarly, constant reflectance at 1.6 µm for the cGST phase of the material.

**Figure 9:**
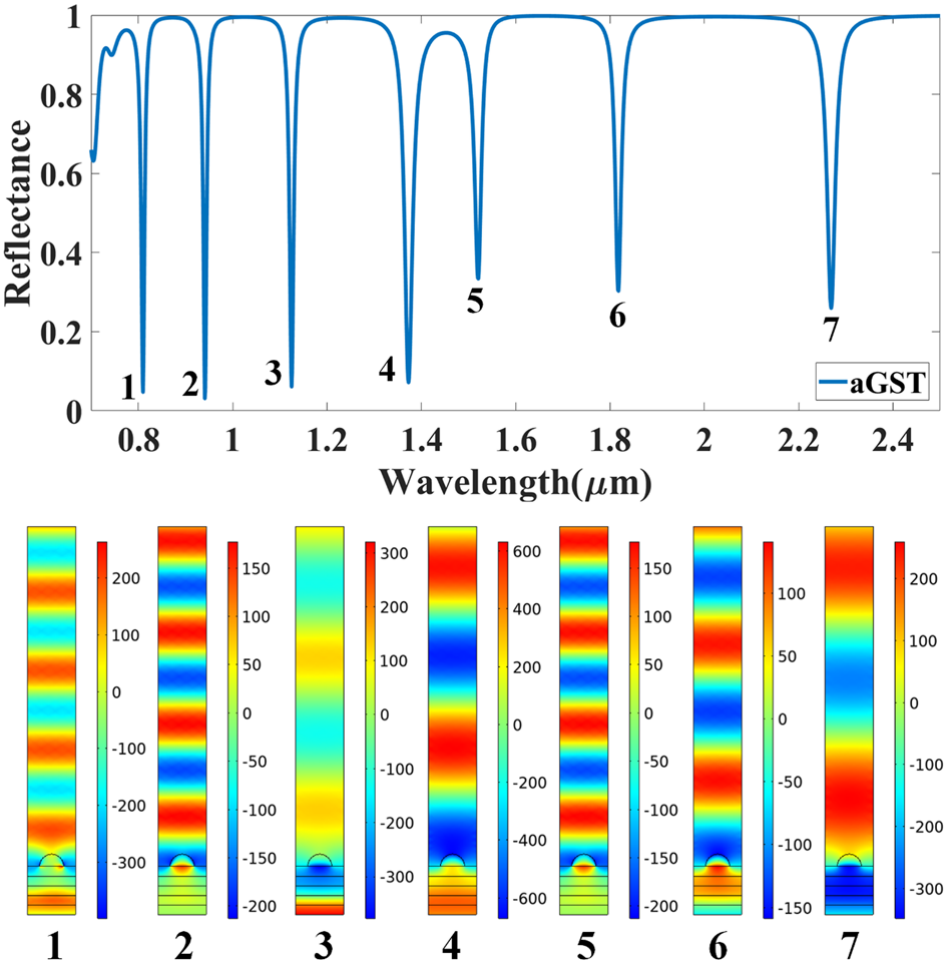
Changes in the electric field component E_z_ for the different reflectance dips of the aGST phase of the proposed SPR sensor. The value of the refractive index is considered 1.34.

**Figure 10:**
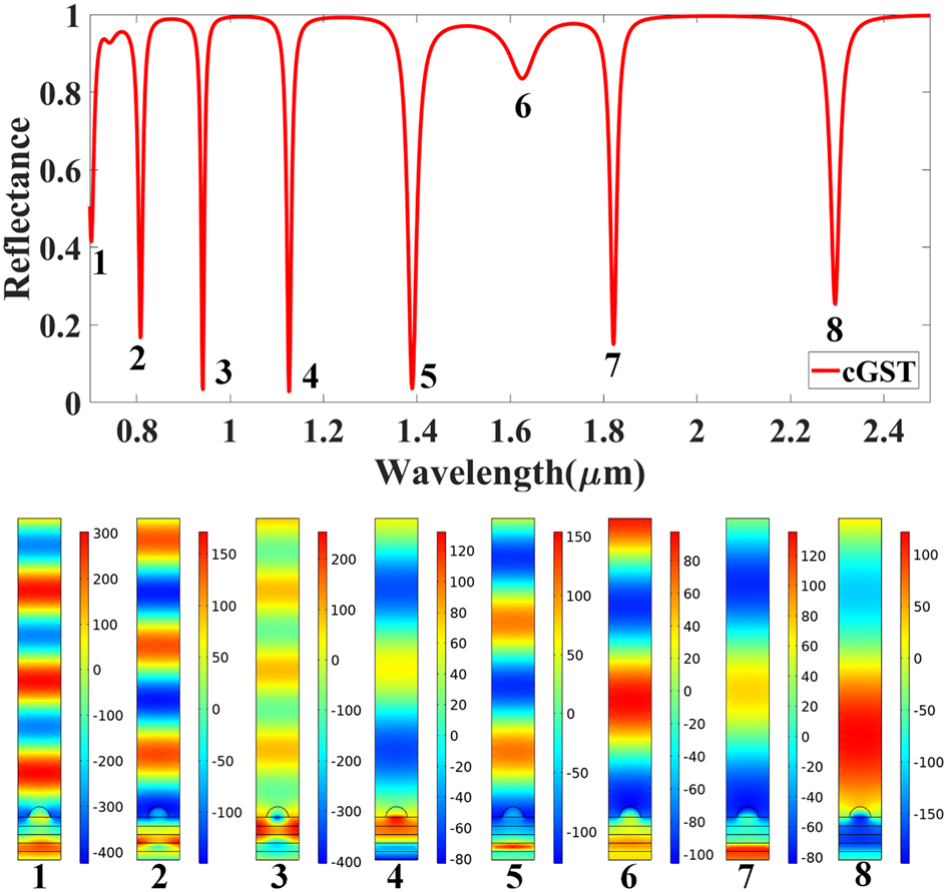
Changes in the electric field component E_z_ for the different reflectance dips of the cGST phase of the proposed SPR sensor. The value of the refractive index is considered 1.34.

**Figure 11:**
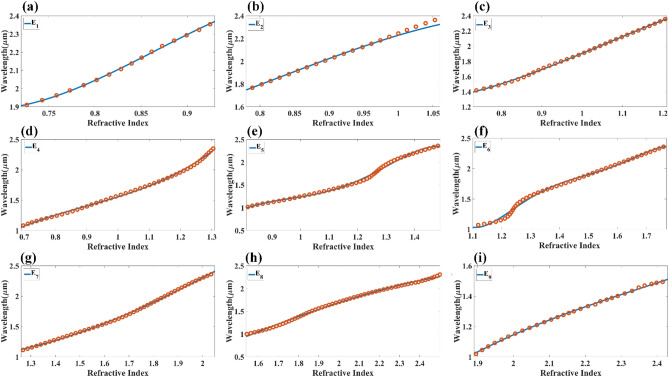
Calculated quadratic fitting curve for the equation (**a–i**) E_1_–E_9_ derived for changes in resonating wavelength peak and its associated refractive index for aGST phase of the material. The multi-layered structure formed in this response is analyte–Ag–Si–GST–Si.

**Figure 12:**
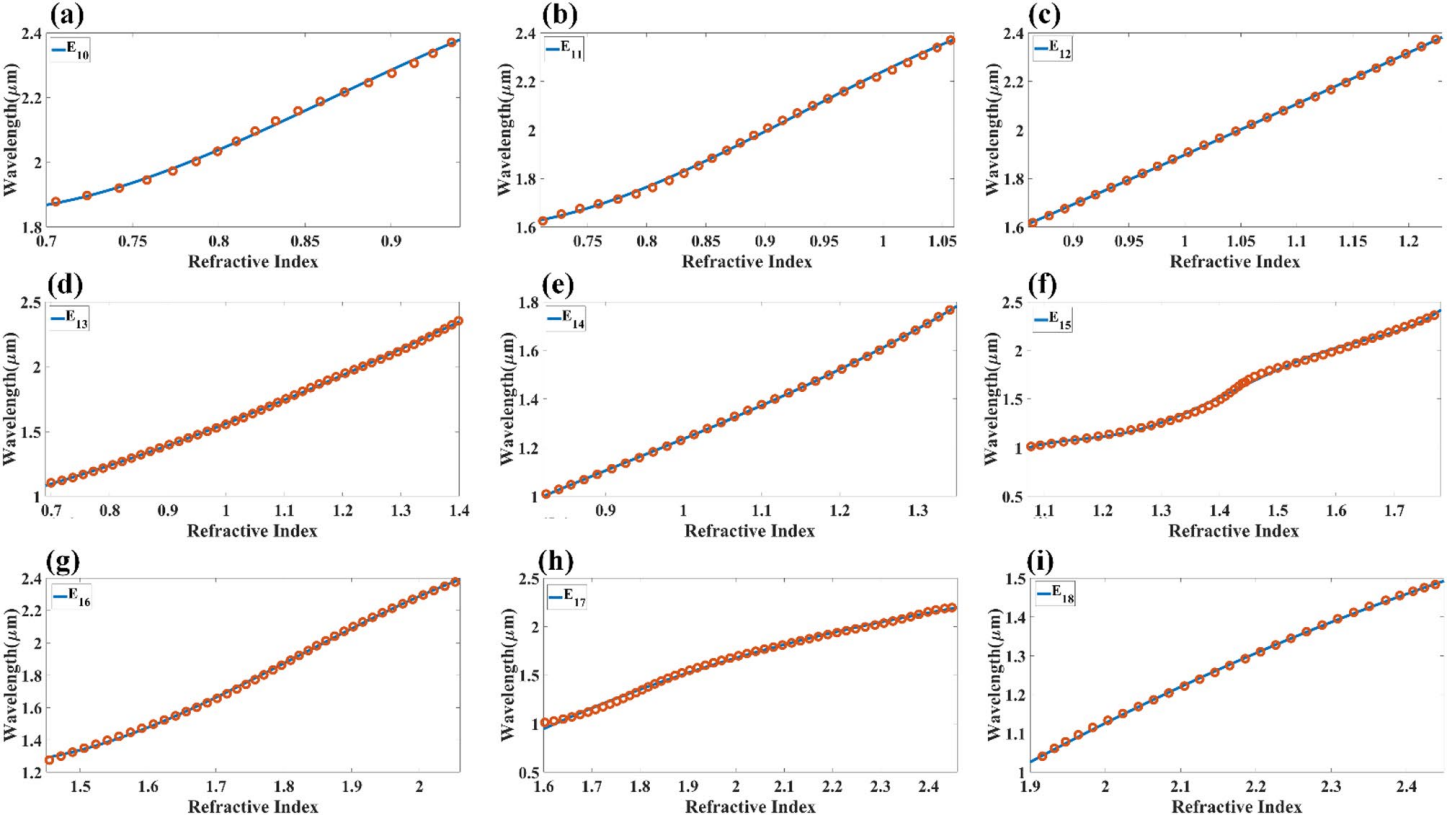
Calculated quadratic fitting curve for the equation (**a–i**) E_10_–E_98_ derived for changes in resonating wavelength peak and its associated refractive index for the cGST phase of the material. The multi-layered structure formed in this response is analyte–Ag–Si–GST–Si.

**Figure 13:**
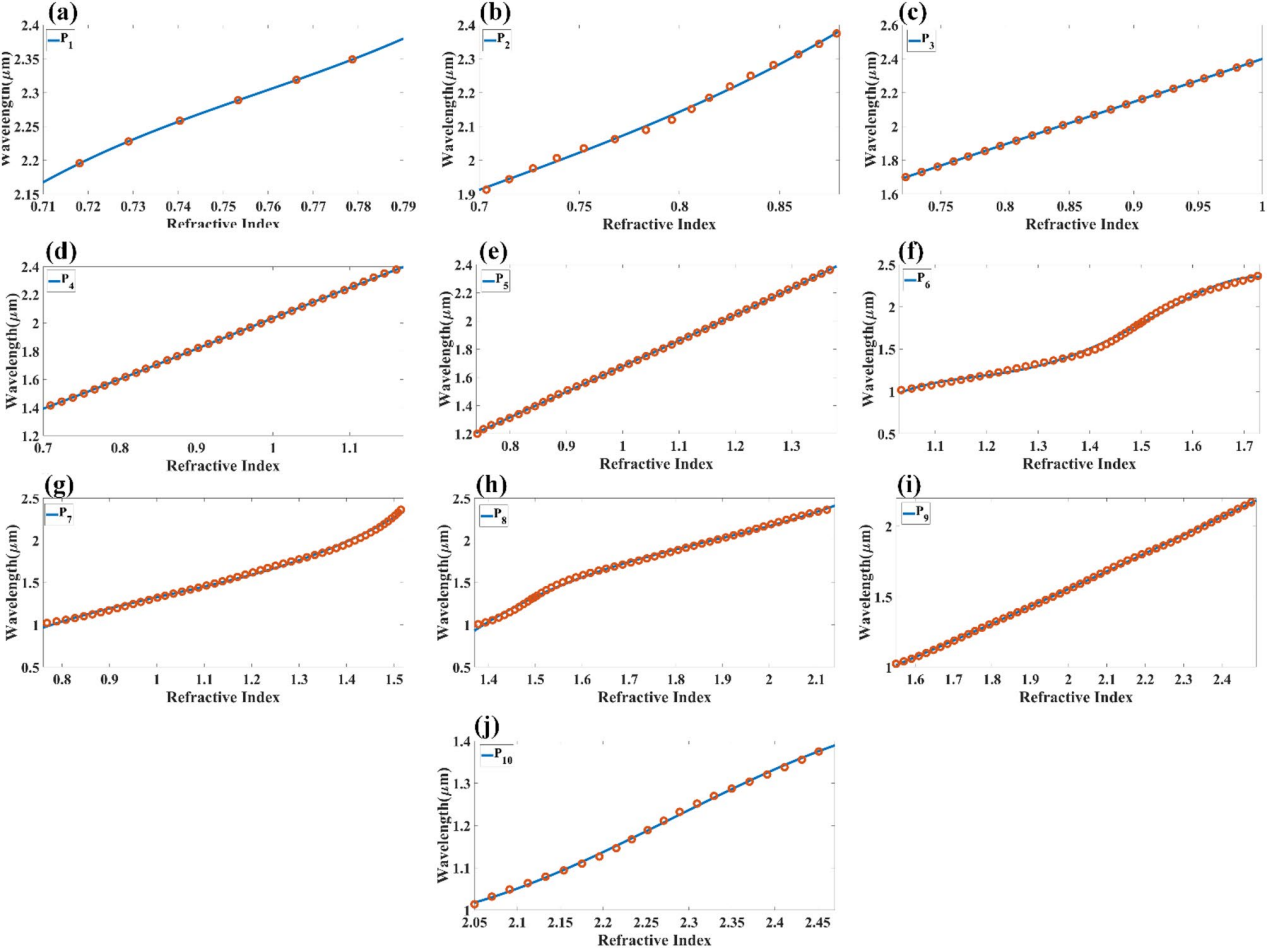
Calculated quadratic fitting curve for the equation (**a–i**) P_1_–P_10_ E_98_ derived for changes in resonating wavelength peak and its associated refractive index for aGST phase of the material. The multi-layered structure formed in this response is analyte–Ag–Si–HfO_2_–Si–GST–Si.

**Figure 14:**
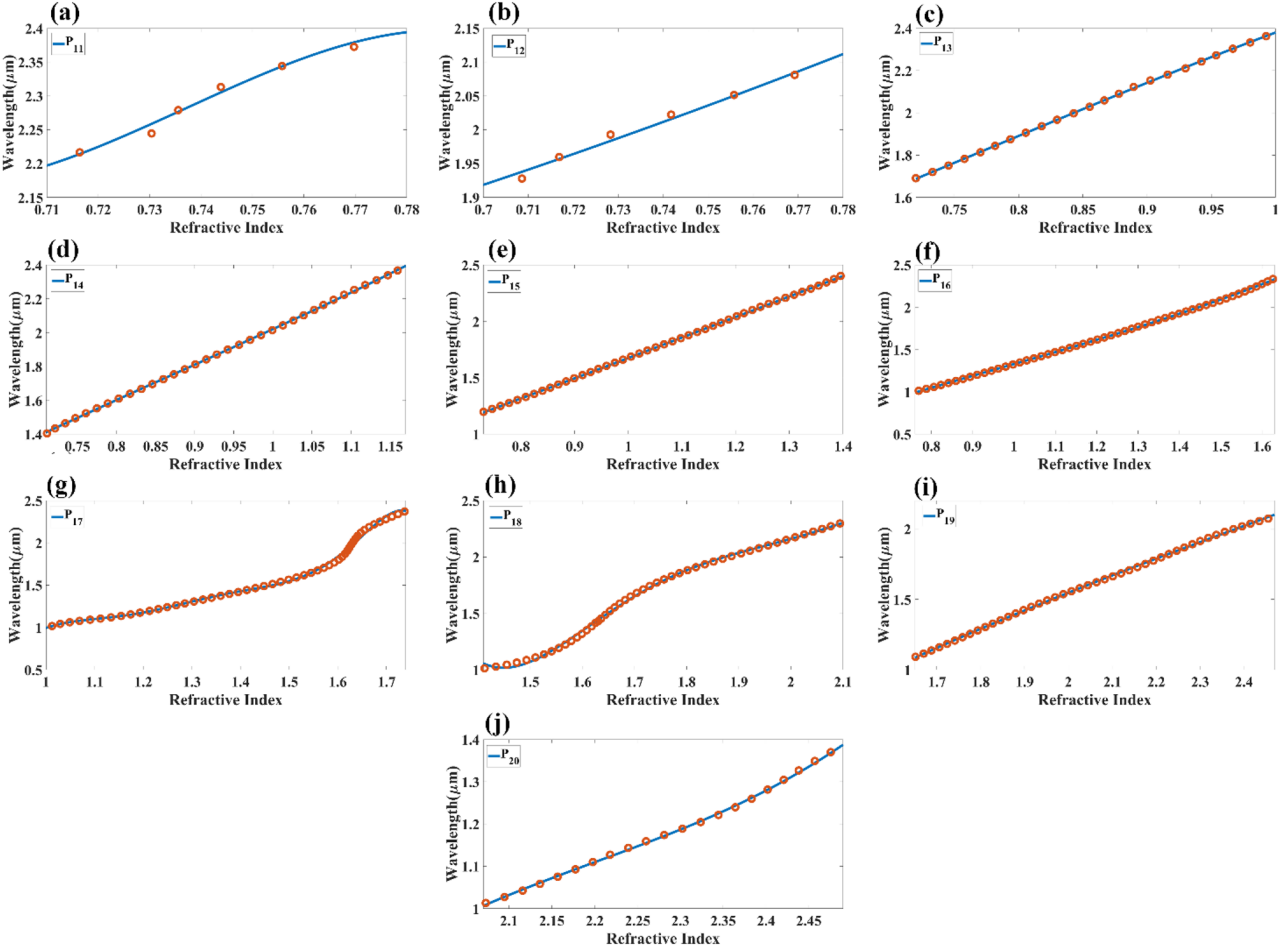
Calculated quadratic fitting curve for the equation (**a–i**) P_11_–P_20_ E_98_ derived for changes in resonating wavelength peak and its associated refractive index for the cGST phase of the material. The multi-layered structure formed in this response is analyte–Ag–Si–HfO_2_–Si–GST–Si.

Figures 9 and 10 show the normalized electric field responsible for the aGST and cGST phases of the analyte–Ag–Si–HfO_2_–Si–GST–Si layered structure. These figures are simulated with the finite element method in COMSOL multiphysics software with periodic boundary mode analysis and port conditions. Figure 9 shows the resonating points at 0.84 µm, 0.94 µm, 1.125 µm, 1.373 µm, 1.52 µm, 1.82 µm, and 2.26 µm for the aGST phase. Similarly, Fig. 10 shows the different resonating points of 0.70 µm, 0.80 µm, 0.94 µm, 1.12 µm, 1.39 µm, 1.62 µm, 1.82 µm, and 2.295 µm for cGST phase of the material. The effect of the normalized electric field interference differs for the different points. The energy concentration for the different layered structures differs for specific resonating peaks. In all the cases, the top Ag layer worked as resonating dipole to trap the specific wavelength, which will differ depending on the analyte placed on the top of the structure. We have identified the fitting curves for the proposed refractive index sensors for all the equation traces P_1_–P_20_ and E_1_–E_18_. The comparative curve of the equation and its tracing curve are shown in Figs. 11 and 12 for the aGST and cGST phases of the Analyte–Ag–Si–GST–Si layered structure. Similarly, Figs. 13 and 14 for the aGST and cGST phases of analyte–Ag–Si–HfO_2_–Si–GST–Si layered structure. It is identified that all the calculated curve equations are fitted with the trace points of reflectance values over the specific range of the wavelength and refractive index.

Refractive indices of the different biomolecules are considered from the reference database provided in ^47–51^. As per the previously published literature, the refractive indices of water, ethanol, blood plasma, urine, glucose, hemoglobin, biotin, saliva, and sweat range from 1 to 2.5. The resonant wavelengths are those that provide maximum transmittance i.e. resonance peaks. The essential concept of sensing for a RI sensor is that if the refractive index or any of the spatial dimensions of the sensor change, the resonant wavelengths will shift to a longer or shorter wavelength region, leading to the identification of the unknown material. The detection of an unknown analyte using SPR biosensors can also be performed based on changes in the wavelength of reflected light rather than the angle of incidence. Here are the steps involved in this method:

- Preparation of the sensing surface: a thin metal film or grating is deposited on a glass or quartz substrate, and a linker molecule is attached to the metal surface. The linker molecule can immobilize a specific receptor, such as an antibody, that selectively binds to the analyte of interest.
- Introduction of the unknown analyte: the sample containing the unknown analyte is introduced to the sensing surface. The analyte can interact with the receptor and cause a change in the refractive index of the sensing surface.
- Measurement of the reflected light spectrum: a broadband light source is used to illuminate the sensing surface, and the spectrum of reflected light is measured using a spectrometer. The reflected light spectrum contains a characteristic dip, called the plasmon dip, at a specific wavelength.
- Detection of the analyte: as the refractive index of the sensing surface changes due to the interaction with the analyte, the wavelength of the plasmon dip shifts. The amount of shift is proportional to the change in the refractive index of the sensing surface, which in turn depends on the concentration of the analyte.
- Quantification of the analyte: the concentration of the analyte can be determined by comparing the measured shift in the wavelength of the plasmon dip to a calibration curve obtained using known concentrations of the analyte.
- Overall, detecting an unknown analyte using SPR biosensors based on changes in the wavelength of reflected light involves measuring the shift in the wavelength of the plasmon dip as the refractive index of the sensing surface changes due to the interaction with the analyte. The concentration of the analyte can be determined by comparing the measured shift to a calibration curve ^45–48,52,53^.

We can also identify the value of the sensitivity of the refractive index sensor proposed in these equation traces by applying the differential formula of $S=d\lambda /dn$. We can identify from the different trace equations that the curve's slope is primarily affected by the sensitivity variation. In a linear curve, the sensitivity variation is constant at the overall range, as observed in various curves (for example, P_12_, P_14_, P_15_). The abrupt sensitivity variation can be observed for curves like P_6_, P_17,_ and P_18_. A similar sensitivity variation can be observed in the E_1_ to E_18_ curve. The sensitivity variation in the E6 curve ranges between 132 and 1240 nm/RIU. The sensitivity variation is slight at the abrupt change between 1.2 and 1.3 µm of the wavelength, which is 132 nm/RIU. In other ranges, the sensitivity is higher where the linear changes between refractive index and wavelength values. This research enables us to choose the operational refractive index range for specific biomolecule samples, and This is because the range of RI values that we may pick from is expanded. The planned structure may also be fine-tuned with the help of the two separate phases of the GST material, referred to as aGST and cGST. Both of these phases are sensitive to fluctuations in temperature. Equations are built by exploiting the structure's adjustable behavior to compute the structure's global reflectance. It is possible to alter the behaviour of the overall refractive index sensor.

## Conclusion

We have shown the results of numerical studies of a phase transition material and a HfO_2_-based visible and infrared wavelength-operated refractive index sensor for a wide range of biomolecule detection. Three different layered designs are explored to learn more about the suggested architecture; each has layers of supplementary materials such as HfO_2_, GST, silver, and silica. We calculated the multilayer structure's reflectance response using a refractive index spectrum from 1.2 to 2.4. Furthermore, we have looked at how the height of the various materials affects the performance of the construction as a whole. We have presented the effect of the different materials with their height parameters to identify the optimized performance of the sensor. We have calculated various resonant trace equations using different resonating points, wavelength points, and refractive index values that may be used to determine the sensing behaviour for a particular wavelength range and refractive index values. These equations can be found in our article. We have created a total of 20 equations in analyte–Ag–Si–HfO_2_–Si–GST–Si layered structure to assist in determining the behaviour of the proposed sensor over a specific range of refractive index and wavelength spectrum. We have also calculated that we have created 18 equations in the analyte–Ag–Si–GST–Si layered structure. The temperature can bring about transitions between aGST and cGST phases of the GST material, which may then be used to fine-tune the suggested structure. A biosensor capable of detecting a large variety of biomolecules can be built with the help of the recommended structure. These biomolecules can be seen in glucose, cholesterol, hemoglobin, urine, and salivary cortisol.

## Data Availability

Data available based upon reasonable request from corresponding author.
